# Supplementary material for: Circulating extracellular vesicle microRNAs mediate immune modulation of social behavior in male mice
Source: Nat Commun. 2026 Apr 3;17:4762. doi: 10.1038/s41467-026-70469-1 (PMC13216276; doi:10.1038/s41467-026-70469-1)

Supplementary Information

Circulating extracellular vesicle microRNAs mediate immune modulation of social behavior in male mice

Ken Matoba[1,9], Eisuke Dohi[1,2,7,9], Phoebe A. Garcia[1,9], Jose Francis-Oliveira[1,9], Mirmohammadali Mirramezanializamini[1], Inssaf Berkiks[1], Frida Anguiano[1], Jana H. Badrani[1] , Oluwaseun Fatoba[1], Eric Y. Choi[2], Julia See[2], Md. Sorwer Alam Parvez[1] , Takahiro Kochi[1], Norimichi Ito[1], Rei Mitani[2], Indigo V.L. Rose[2], Takashi Imai[2], David K. Crossman[3], Mikhail V. Pletnikov[2,8], Kenneth W. Witwer[4], Minae Niwa[1,2,5,6], and Shin-ichi Kano[1,2,5]*

[1]Department of Psychiatry and Behavioral Neurobiology, University of Alabama at Birmingham Heersink School of Medicine, Birmingham, AL, USA
[2]Department of Psychiatry and Behavioral Sciences, Johns Hopkins University School of Medicine, Baltimore, MD, USA
[3]Department of Genetics, University of Alabama at Birmingham Heersink School of Medicine, Birmingham, AL, USA
[4]Departments of Molecular and Comparative Pathobiology and Neurology, and The Richman Family Precision Medicine Center of Excellence in Alzheimer's Disease, Johns Hopkins University School of Medicine, Baltimore, MD, USA
[5]Department of Neurobiology, University of Alabama at Birmingham Heersink School of Medicine, Birmingham, AL, USA
[6]Department of Biomedical Engineering, University of Alabama at Birmingham School of Engineering, Birmingham, AL, USA
[7]Present address: National Institute of Neuroscience, National Center of Neurology and Psychiatry, Tokyo, Japan
[8]Present address: Department of Physiology and Biophysics, Jacobs School of Medicine and Biomedical Sciences, SUNY University at Buffalo, Buffalo, NY, USA
[9]These authors equally contribute to this work.

*Correspondence should be addressed to S.K. (sikano@uabmc.edu)

Supplementary Fig. 1

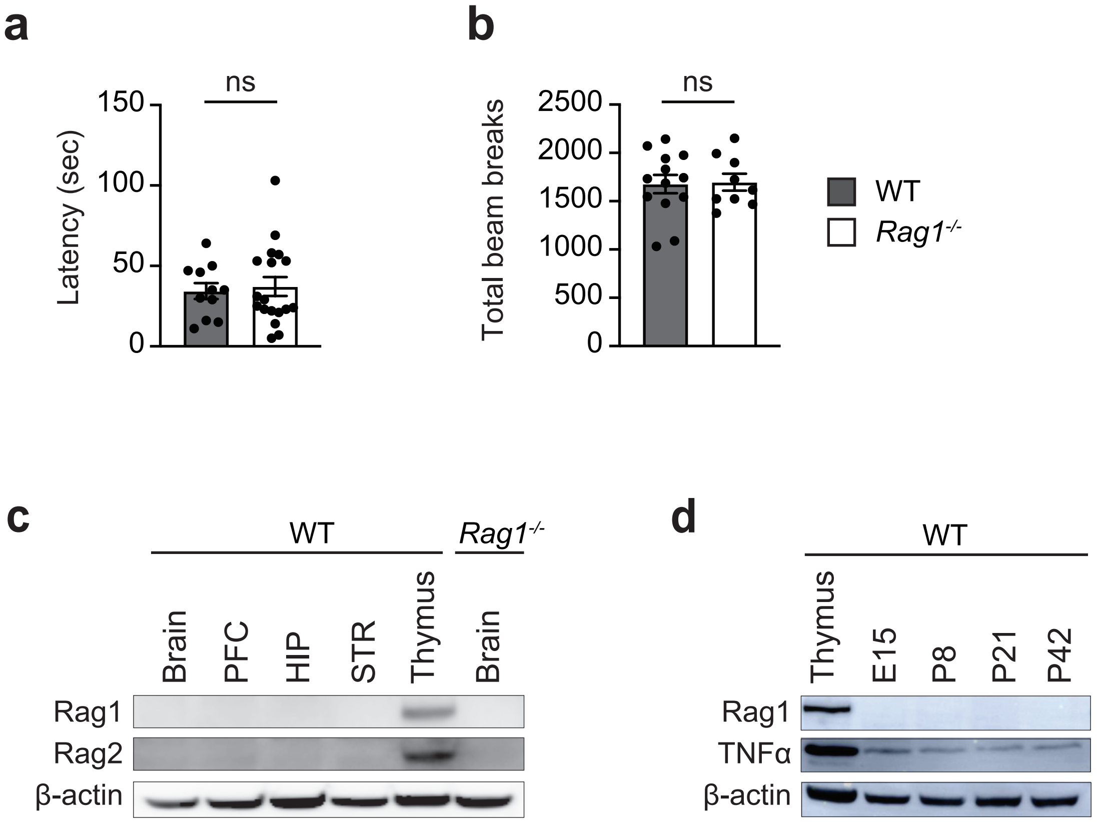

Supplementary Fig. 1: Additional behavioral data of *Rag1*$^{-/-}$ mice and Rag protein expression in the brain.

a, No difference in latency to find a buried food pellet in the buried food pellet test between WT and *Rag1*$^{-/-}$ mice. WT mice, n = 11; *Rag1*$^{-/-}$ mice, n = 18. **b**, No difference in total activities analyzed with the open field test between WT and *Rag1*$^{-/-}$ mice. WT mice, n = 13; *Rag1*$^{-/-}$ mice, n = 9. **c**, Western blot images showing no detectable Rag1 and Rag 2 proteins in the brains of WT mice. PFC, prefrontal cortex; HC, hippocampus; STR, striatum. **d**, Western blot images showing Rag1, TNFα, and β-actin proteins in the thymus and brains of WT mice (embryonic day 15 (E15), postnatal day 8 (P8), P21, and P42. Rag1 and TNFα images were taken with the same exposure time. Each bar graph represents mean ± SEM. Each dot represents one mouse. ns, not significant. Significance was determined by Student's *t*-test. See Supplementary Data 13 for details of the statistical analysis. Source data are provided as a Source Data file.

Supplementary Fig. 2

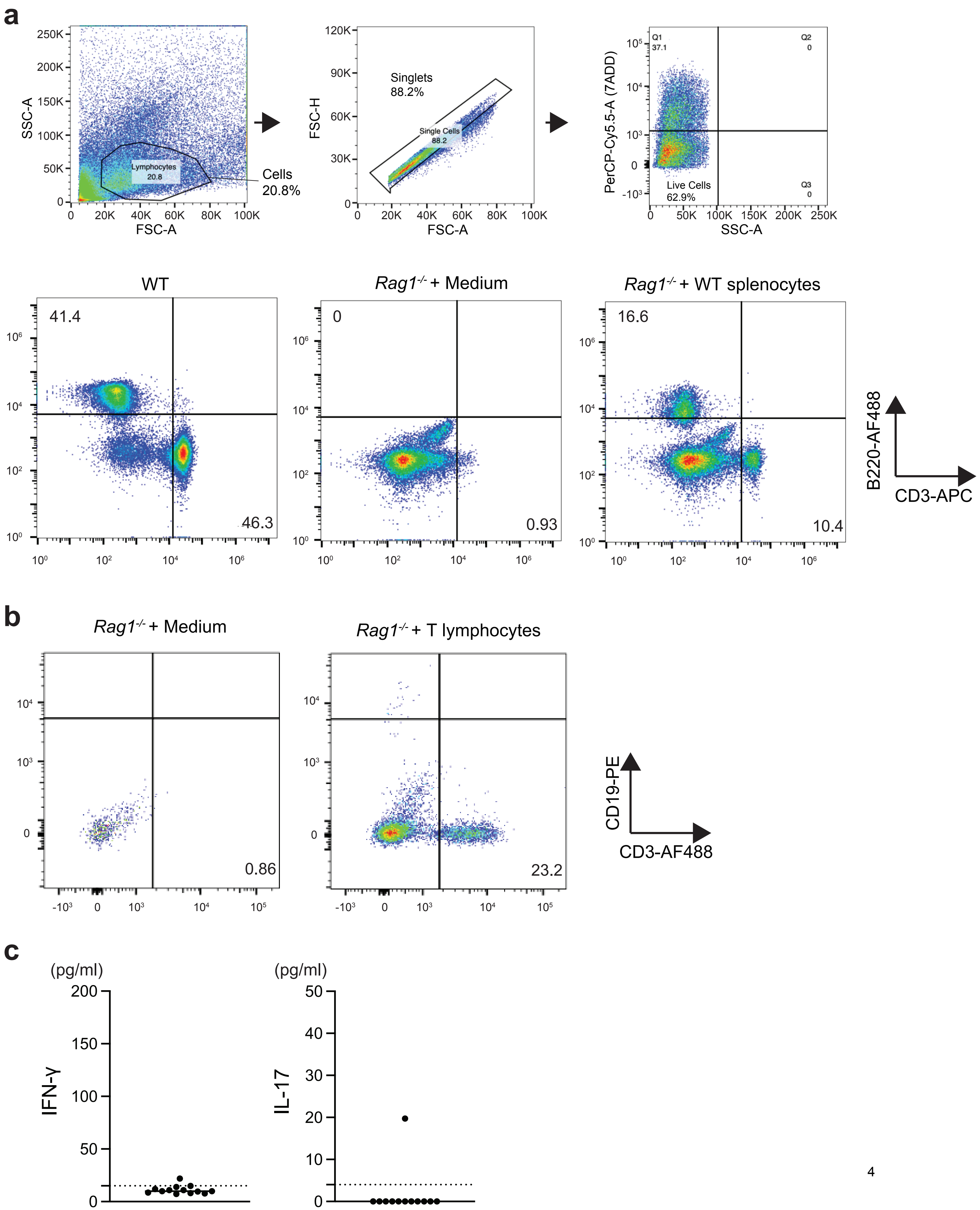

Supplementary Fig. 2: Lymphocyte reconstitution after adoptive cell transfer

a, Flow cytometry data showing the recovery of $CD3^+$ T cells and $B220^+$ B cells in *Rag1*$^{-/-}$ mice adoptively transferred with WT splenocytes. Percentages shown in the plots are calculated based on the parent gate. **b**, Flow cytometry data showing the recovery of $CD3^+$ T cells in *Rag1*$^{-/-}$ mice adoptively transferred with WT T cells. **c**, ELISA data showing the levels of IFN-γ and IL-17 in WT mouse sera. Source data are provided as a Source Data file.

Supplementary Fig. 3

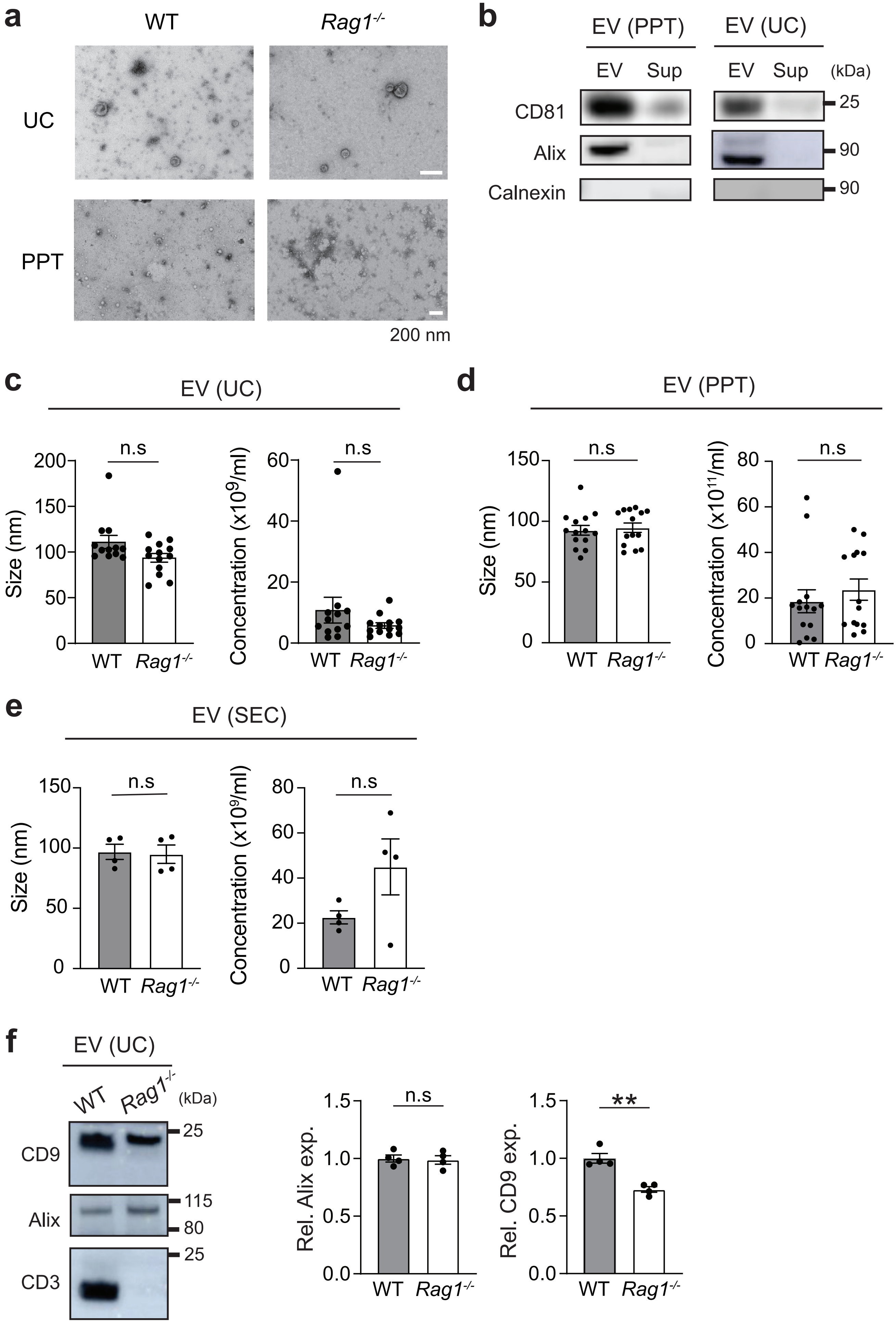

Supplementary Fig. 3: Characterization of EVs prepared with different methods.

a, Representative transmission electron microscopy (TEM) images of EVs prepared with differential ultracentrifugation (UC) and precipitation (PPT). Scale bar = 200 nm. **b**, Western blot images of EV marker proteins, CD81 and Alix, in EVs prepared with PPT and UC. Calnexin, a cytoplasmic protein marker. Sup, supernatants after EV precipitations. **c**, Nanoparticle tracking analysis of bEVs from WT and $Rag1^{-/-}$ mice using NanoSight NS300. WT, n = 12; $Rag1^{-/-}$, n = 13. **d**, NanoSight® analysis of bEVs prepared with PPT from WT and $Rag1^{-/-}$ mouse sera. WT, n = 14; $Rag1^{-/-}$, n = 14. **e**, ZetaView® analysis of EVs prepared with size exclusion chromatography (SEC) from WT and $Rag1^{-/-}$ mouse sera. WT, n = 4; $Rag1^{-/-}$, n = 4. **f**, Expression levels of EV marker proteins, CD9 and Alix, in WT and $Rag1^{-/-}$ samples (n=4 per group). Left, representative Western blot images. Right, quantification graphs showing the relative expression levels. Equal amounts of proteins were loaded into each lane. Each bar graph represents mean ± SEM. Each dot represents one mouse. $^{**}p < 0.01$. n.s., not significant. Significance was determined by Mann-Whitney U test and Student's *t*-test. See Supplementary Data 13 for details of the statistical analysis. Source data are provided as a Source Data file.

Supplementary Fig. 4

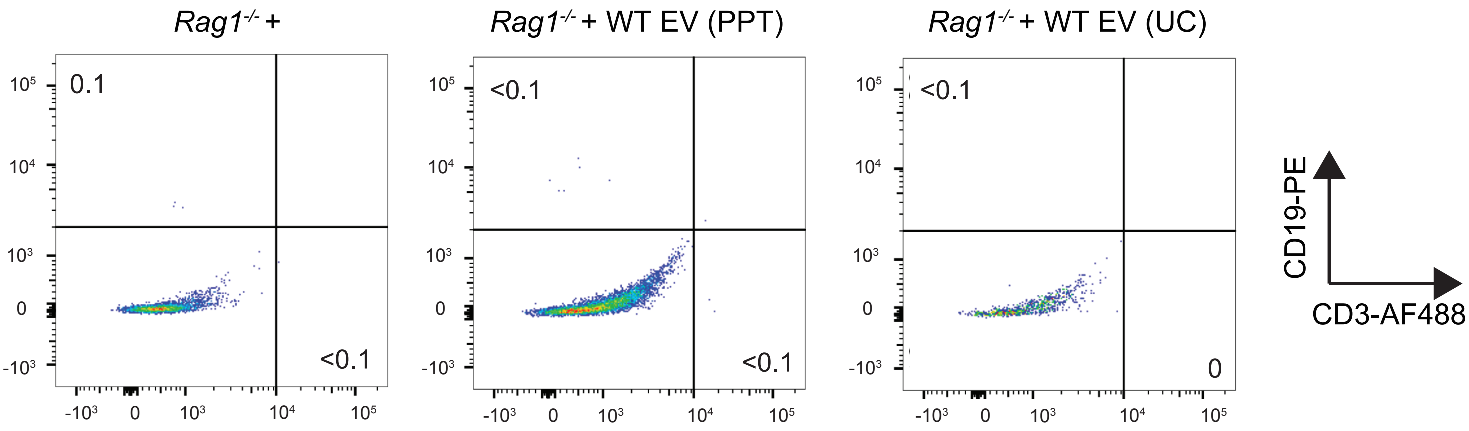

Supplementary Fig. 4: No recovery of T and B cells in *Rag1$^{-/-}$* mice intravenously injected with bEVs from WT mice.

No changes in T or B cell populations were detected in the spleen from *Rag1$^{-/-}$* mice after intravenous injection with bEVs from WT mice. Source data are provided as a Source Data file.

Supplementary Fig. 5

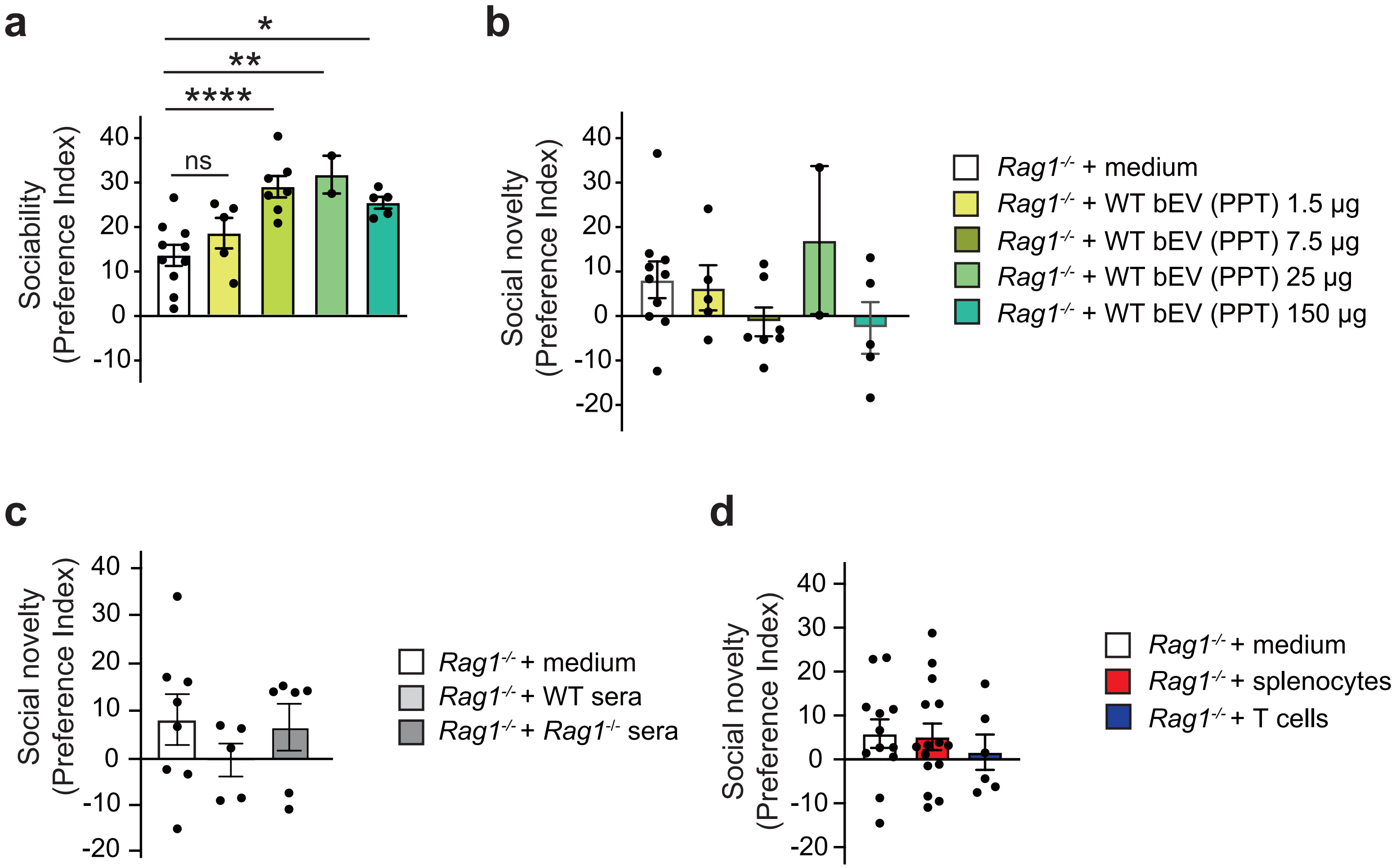

Supplementary Fig. 5: No recovery in social novelty preference by *Rag1$^{-/-}$* mice after intravenous injection with WT bEVs or adoptive transfer of WT splenocytes or T cells.
a, b, Intravenous injection of WT bEVs (enriched by PPT) into *Rag1$^{-/-}$* mice increased their sociability, but not social novelty preference. *Rag1$^{-/-}$* mice + medium, n = 10; *Rag1$^{-/-}$* mice + WT bEVs 1.5 μg, n = 5; *Rag1$^{-/-}$* mice + WT bEVs 7.5 μg, n = 7; *Rag1$^{-/-}$* mice + WT bEVs 25 μg, n = 2; and *Rag1$^{-/-}$* mice + WT bEVs 150 μg, n = 5. **c**, Intravenous injection of WT and *Rag1$^{-/-}$* sera into *Rag1$^{-/-}$* mice did not show any beneficial effect on their social novelty preference deficits. *Rag1$^{-/-}$* mice + medium, n = 8; *Rag1$^{-/-}$* mice + WT sera, n = 5; *Rag1$^{-/-}$* mice + *Rag1$^{-/-}$* sera, n = 6. **d**, Adoptive transfer of neither splenocytes nor T cell reconstitution on *Rag1$^{-/-}$* mice showed any beneficial effect on their social novelty preference deficits. *Rag1$^{-/-}$* mice + medium, n = 12; *Rag1$^{-/-}$* mice + WT splenocytes, n = 15; *Rag1$^{-/-}$* mice + WT T cells, n = 6. Each bar graph represents mean ± SEM. Each dot represents one mouse. ns, not significant. **p* < 0.05, ***p* < 0.01, *****p* < 0.001. ns, not significant. Significance was determined by one-way ANOVA with post-hoc Dunnett's test. See Supplementary Data 13 for details of the statistical analysis. Source data are provided as a Source Data file.

Supplementary Fig. 6

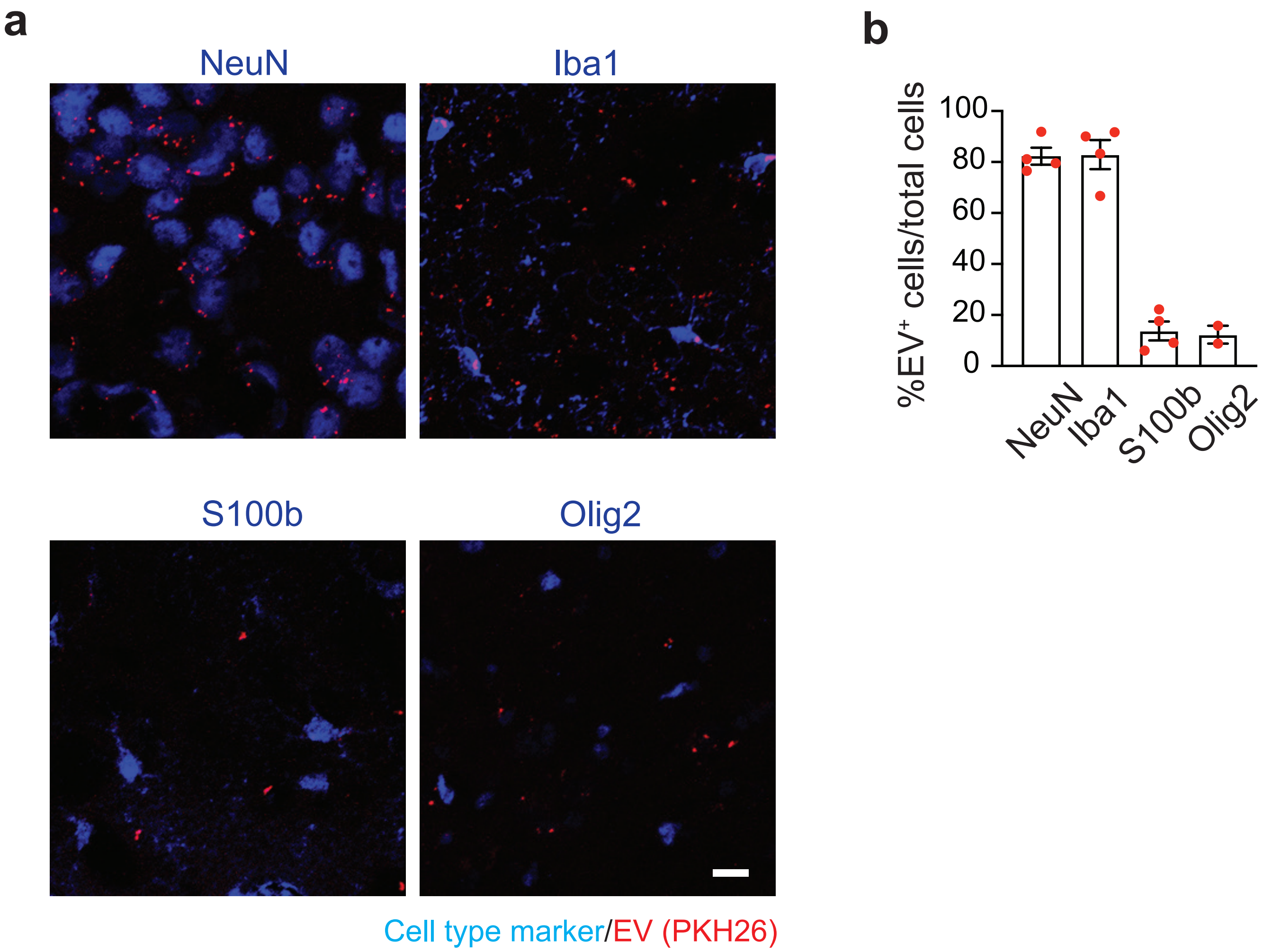

Supplementary Fig. 6: Co-localization of PKH26-labeled bEVs with neurons and microglia.

a, Representative confocal microscope images showing the localization of PKH26-labeled bEVs with $NeuN^+$ neurons and $Iba1^+$ microglia in the mPFC of *Rag1*$^{-/-}$ mice. **b,** Quantification of PKH26-labeled EVs localized with $NeuN^+$ neurons, $Iba1^+$ microglia, $S100b^+$ astrocytes, and $Olig2^+$ oligodendrocyte-lineage cells in the mPFC of *Rag1*$^{-/-}$ mice, n = 3. Scale bar, 10 μm. Each bar graph represents mean ± SEM. Source data are provided as a Source Data file.

Supplementary Fig. 7

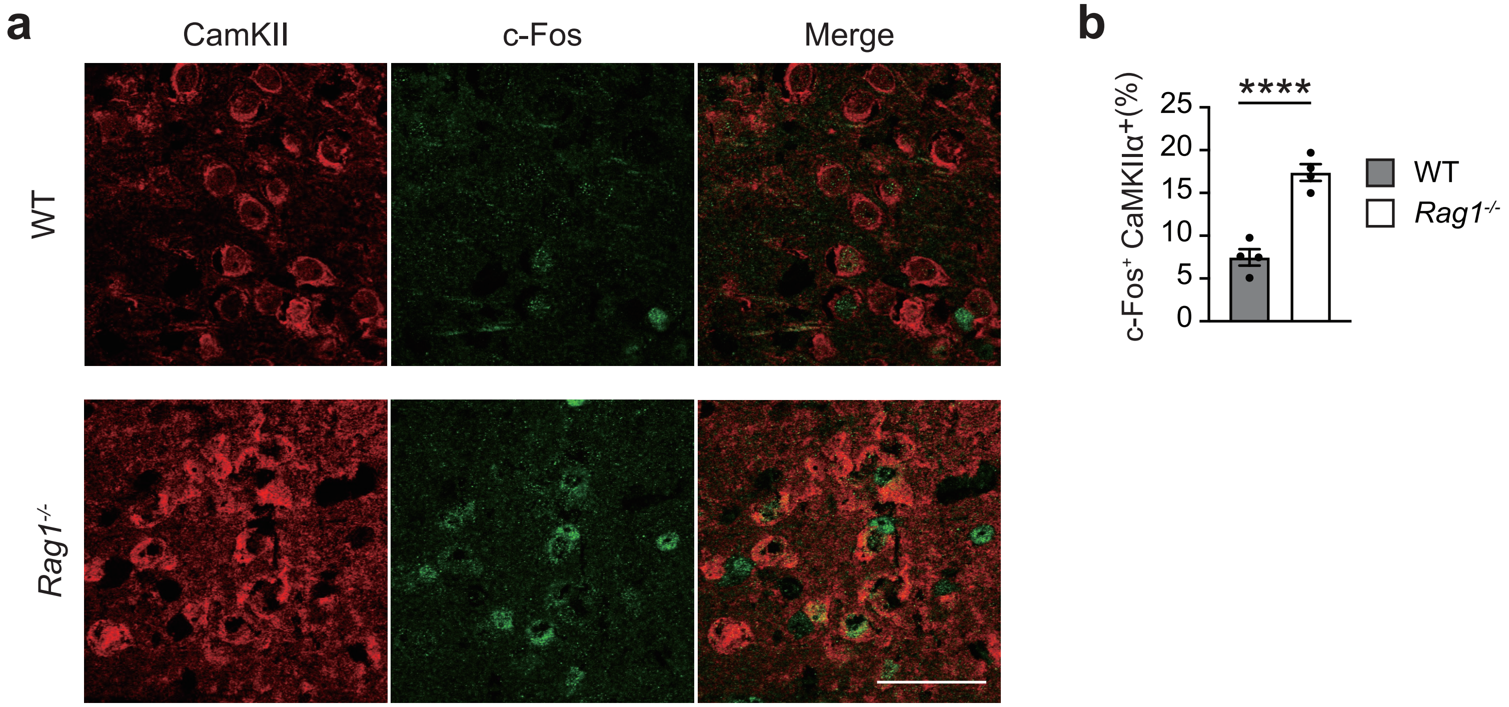

Supplementary Fig. 7: Increased cFos expression in CaMKIIα^{+} neurons in the mPFC of *Rag1*$^{-/-}$ mice.

a, Representative images of c-Fos immunoreactivities in CaMKIIα^{+} neurons in the mPFC of *Rag1*$^{-/-}$ mice compared with WT mice. Scale bar, 50 μm. **b,** Quantification of c-Fos-positive CaMKIIα^{+} neurons as percentages for c-Fos cells per CaMKIIα^{+} neurons. WT mice, n = 4; *Rag1*$^{-/-}$ mice, n = 4. ****p <0.001. ns, not significant. Significance was determined by Student's *t*-test. See Supplementary Data 13 for details of the statistical analysis. Source data are provided as a Source Data file.

Supplementary Fig. 8

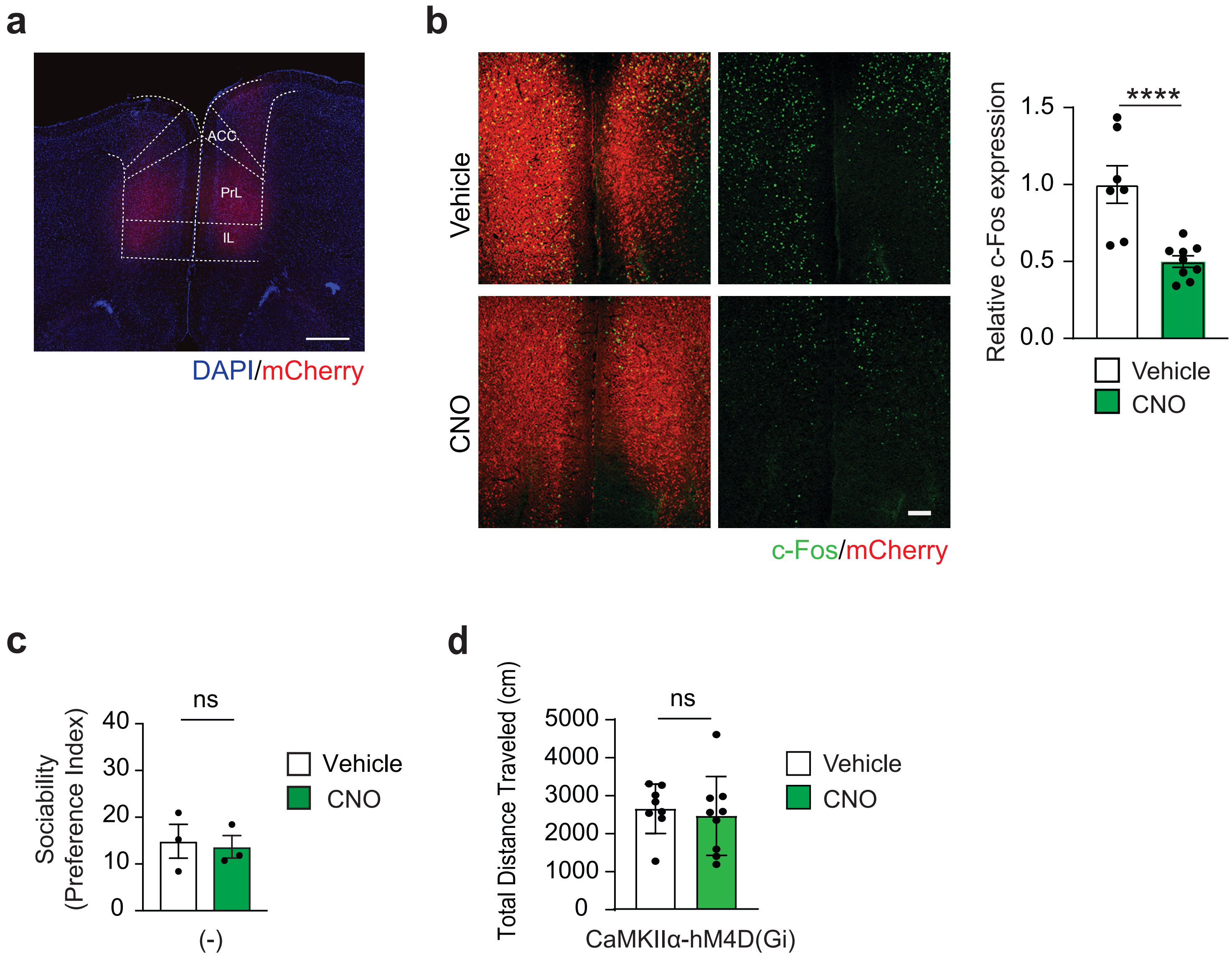

Supplementary Fig. 8: Validation of DREADD experiments for chemogenic inhibition of CaMKIIα^{+} neurons in the mPFC of *Rag1$^{-/-}$* mice.

a, Representative image of AAV-CaMKIIα-hM4D(Gi)-mCherry expression in the mPFC of *Rag1$^{-/-}$* mice. Scale bar, 500 µm. **b**, c-Fos immunoreactivities were suppressed in CNO injected group compared with Vehicle injected group. Vehicle n = 7; CNO n = 8. **c**, No impact of CNO injections on sociability behaviors in *Rag1$^{-/-}$* mice lacking the expression of DREADD construct. Vehicle n = 3; CNO n = 3. **d**, No significant changes in total travel distance during sociability assays in *Rag1$^{-/-}$* mice expressing DREADD construct upon CNO injections. Vehicle n = 8; CNO n = 9. Scale bar, 50 µm. Each bar graph represents mean ± SEM. Each dot represents one mouse. ****$p < 0.01$. ns, not significant. Significance was determined by Student's *t*-test. See Supplementary Data 13 for details of the statistical analysis. Source data are provided as a Source Data file.

Supplementary Fig. 9

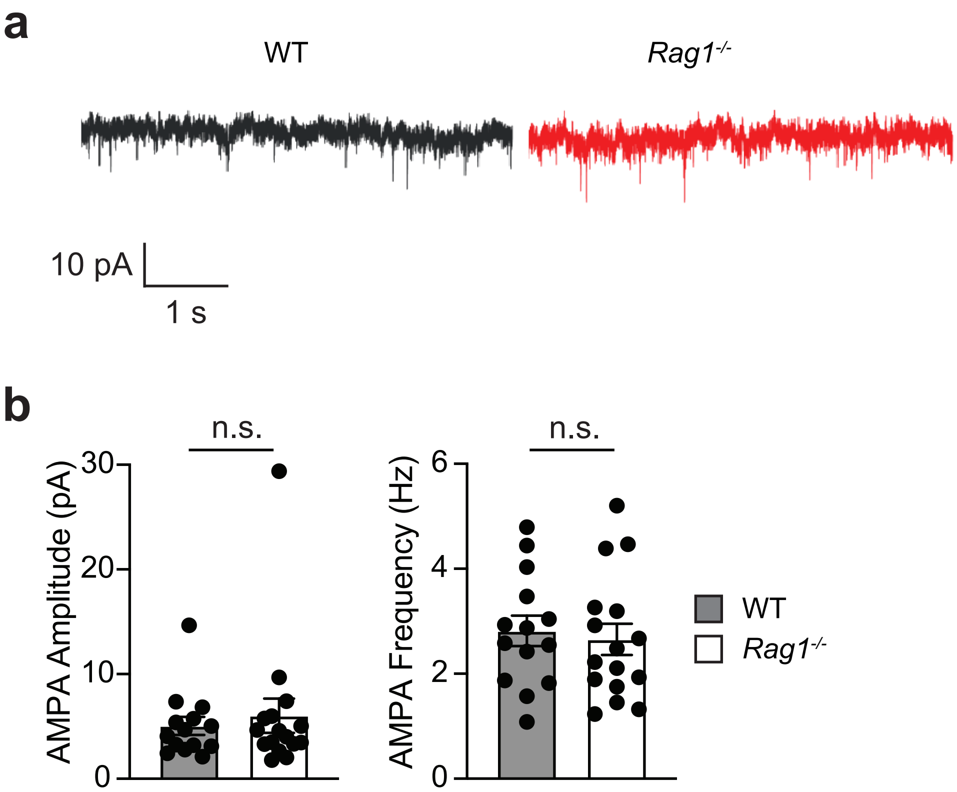

Supplementary Fig. 9: No changes in spontaneous excitatory AMPAR signaling in the mPFC pyramidal neurons of *Rag1*$^{-/-}$ mice.

a, Representative traces of a gap free recording of pyramidal neuros in the mPFC (PrL). Recordings were made in the presence of AP-V (50 μM). sEPSCs through AMPA receptors can be seen. **b,** No significant difference in the amplitudes or the frequencies of AMPA currents between WT and *Rag1*$^{-/-}$ mice. Each bar graph represents mean ± SEM. Each dot represents one cell. n.s., not significant. Significance was determined by Student's *t*-test. See Supplementary Data 13 for details of the statistical analysis. Source data are provided as a Source Data file.

Supplementary Fig. 10

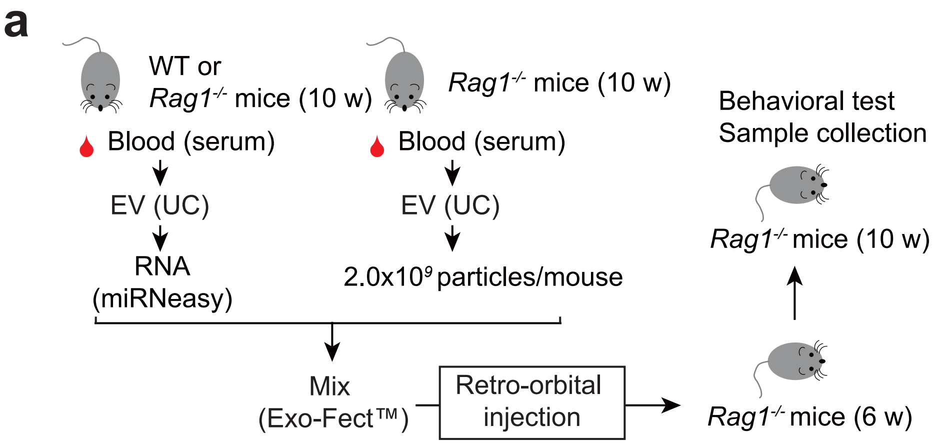

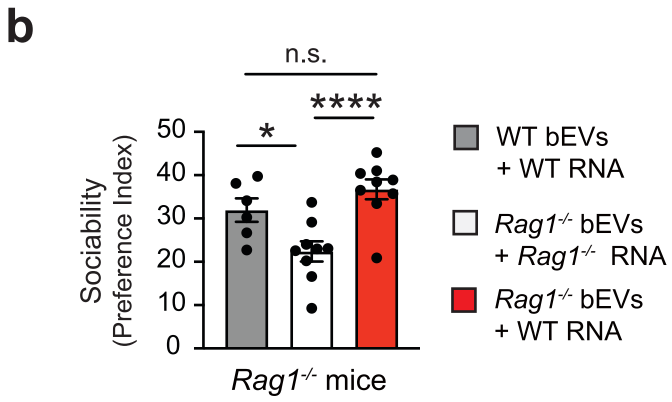

Supplementary Fig. 10: Differential effects of bEV-derived RNAs.

a, Experimental outline of bEV collection, intravenous injection, and subsequent behavioral assay. **b**, Sociability data of *Rag1*$^{-/-}$ mice administered with WT bEVs + WT RNA, *Rag1*$^{-/-}$ bEVs + *Rag1*$^{-/-}$ RNA, and *Rag1*$^{-/-}$ bEVs + WT RNA. WT bEVs + WT RNA, n = 6; *Rag1*$^{-/-}$ bEVs + *Rag1*$^{-/-}$ RNA, n = 9; *Rag1*$^{-/-}$ bEVs + WT RNA, n = 9. Each bar graph represents mean ± SEM. Each dot represents one mouse. $*p < 0.05$, $****p < 0.001$. n.s., not significant. Significance was determined by one-way ANOVA with post-hoc Tukey's test. See Supplementary Data 13 for details of the statistical analysis. Source data are provided as a Source Data file.

Supplementary Fig. 11

a

```
                                      27        34
Prkce 3'UTR       5'...GGAUGGAGGGAGCUCAUGCUGCA...3'
                                      | | | | | | |
mmu-miR-103-3p    3' ...AGUAUCGGGACAUGUUACGACGA...5'

                                     1493      1499
Prkce 3'UTR       5'  ...UAUAGAGGUAUCAACUGCUGCAU...3'
                                       | | | | | |
mmu-miR-103-3p    3'...AGUAUCGGGACAUGUUACGACGA...  5'

                                    2835      2841
Prkce 3'UTR       5' ...ACUGUACUCAAAAGGAUGUGAAU...3'
                                      | | | | | |
mmu-miR-23a-3p    3'  ...CCUUUAGGGACCGUUACACUA...  5'
```

b

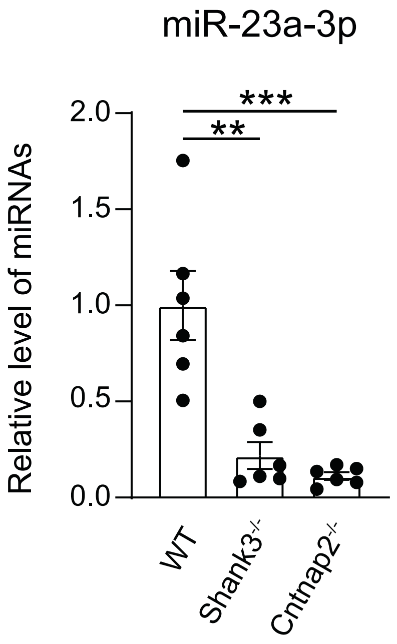

Supplementary Fig. 11: Predicted targeting sites for miR-23a-3p and miR-103-3p in the 3' UTR of *Prkce* gene.

a, Predicted consequential pairings of target region (top) and miRNA (bottom) are shown for miR-23a-3p and miR-103-3p. The target regions are conserved between mouse and human. Prediction data were obtained using TargetScan (release 8.0: September 2021). **b**, Expression of miR-23a-3p in the bEVs from WT, *Shank3*$^{-/-}$, and Cntnap2$^{-/-}$ mice (n = 6 per group). Data are shown as fold-change relative to WT data. Each bar graph represents mean ± SEM. **$p < 0.01$, ***$p < 0.005$. Each dot represents one mouse. Significance was determined by one-way ANOVA with post-hoc Dunnett's test. See Supplementary Data 13 for details of the statistical analysis. Source data are provided as a Source Data file.

Supplementary Fig. 12

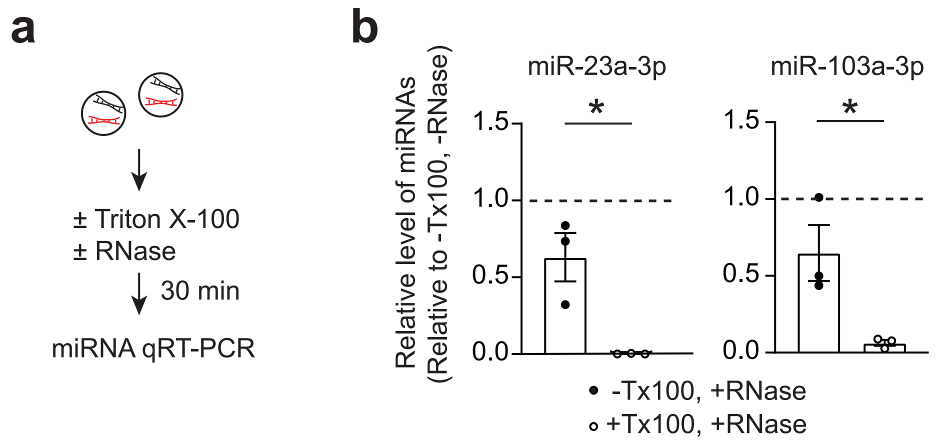

Supplementary Fig. 12: EVs protect miR-23a-3p and miR-103-3p from RNase.

a, Experimental procedures for Triton-X100 and RNase treatments of bEVs. **b**, Quantitative reverse-transcription PCR (qPCR) data of miR-23a-3p and miR-103-3p expression in WT bEV fractions treated as in **a.** Each bar graph represents mean ± SEM. *p<0.05. Each dot represents one mouse. Significance was determined by Student's *t*-test. See Supplementary Data 13 for details of the statistical analysis. Source data are provided as a Source Data file.

Supplementary Fig. 13

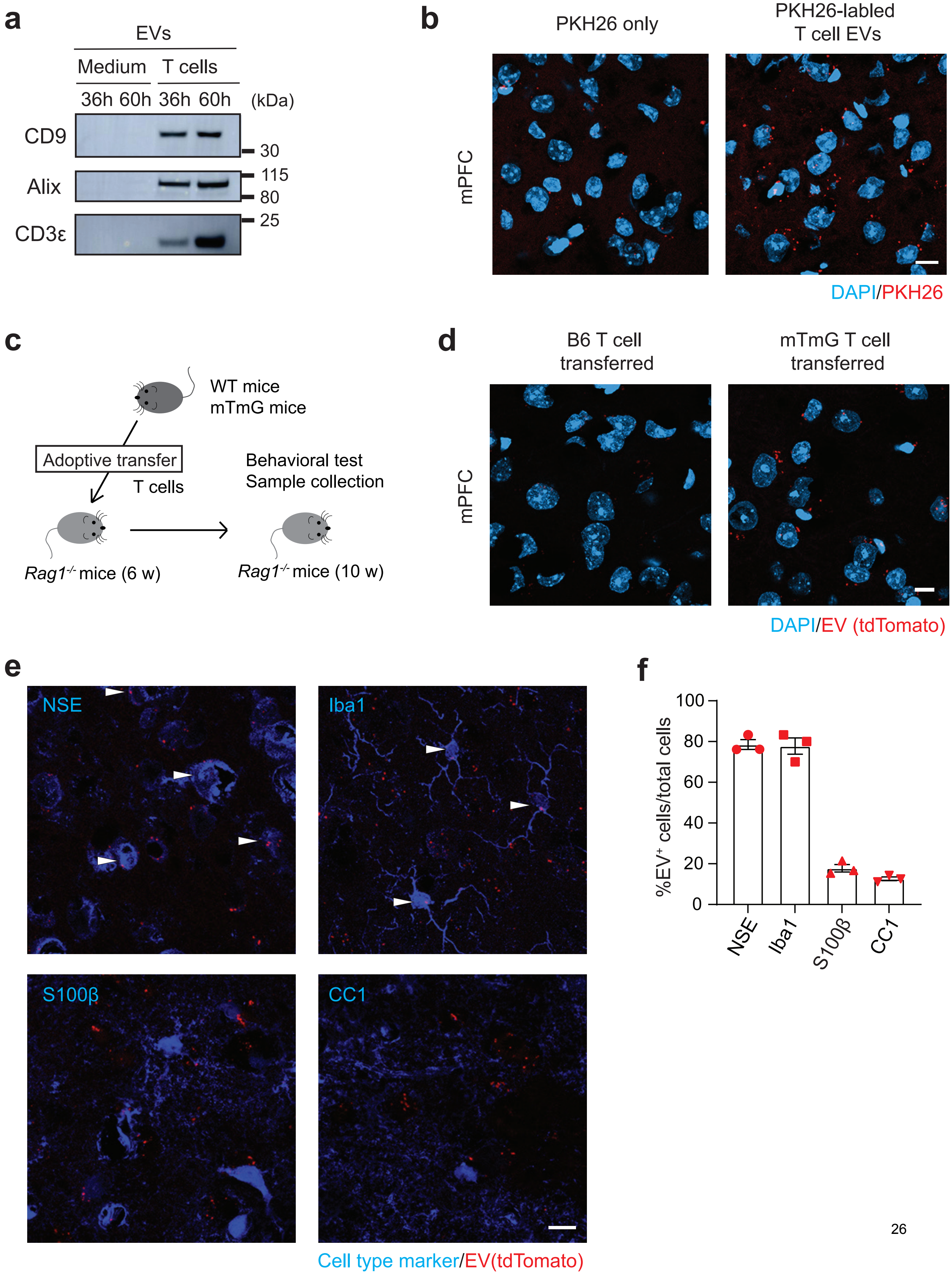

Supplementary Fig. 13: T cell-derived EVs contain miR-23a-3p and co-localize with neurons following intravenous injection.

a, Western blot data of CD9, Alix, and CD3ε protein expression in EVs harvested from T cell culture medium at 36 h and 60 h after incubation. Culture medium without T cells is used as control. **b**, Representative confocal microscope images of PKH26-labeled T cell-derived EVs (red) in the mPFC. PKH26 dye injection without EVs are used as control. **c**, Experimental outline of adoptive transfer/serum injection and subsequent behavioral assay. **d**, Representative confocal microscope images of EVs from tdTomato^{+} T cells (mTmG mouse T cells) adoptively transferred into *Rag1*$^{-/-}$ mice. Immunohistochemistry was conducted 4 weeks after the adoptive transfer. **e**, Representative confocal microscope images of co-localization of tdTomato^{+} T cell-derived EVs (red) and neurons, microglia, astrocytes, and oligodendrocytes in the mPFC of *Rag1*$^{-/-}$ mice. EV^{+} cells are indicated by white arrowheads. **f,** Quantification data of tdTomato^{+} EV co-localization with specific brain cell types (n = 3 mice). Scale bar, 10 μm. Each bar graph represents mean ± SEM. Each dot represents one mouse. See Supplementary Data 13 for details of the statistical analysis. Source data are provided as a Source Data file.

Supplementary Fig. 14

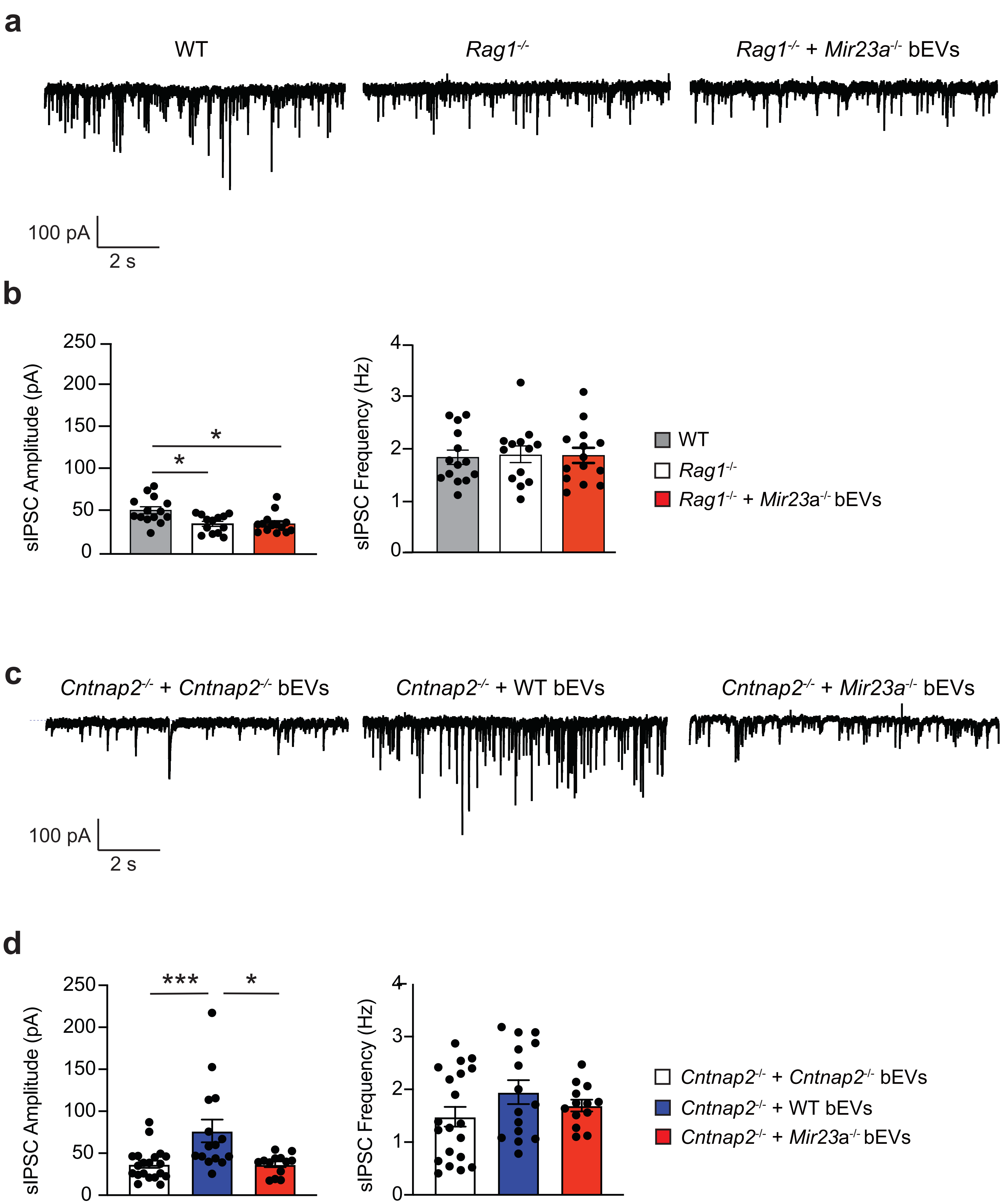

Supplementary Fig. 14: *Mir23a* deficiency impairs the beneficial effects of bEVs on sIPSCs in mPFC pyramidal neurons of *Rag1*$^{-/-}$ and *Cntnap2*$^{-/-}$ mice.

a, Representative traces of sIPSCs in *Rag1*$^{-/-}$ mPFC (PrL) pyramidal neurons exposed to *Mir23a*$^{-/-}$ bEVs, in comparison to those in WT and *Rag1*$^{-/-}$ mPFC. **b,** Quantification of sIPSC amplitude and frequency between groups. **c,** Representative traces of sIPSCs in *Cntnap2*$^{-/-}$ mPFC (PrL) pyramidal neurons exposed to *Cntnap2*$^{-/-}$, WT and *Mir23a*$^{-/-}$ bEVs as described in Fig. 5. *Cntnap2*$^{-/-}$ + WT bEV data are the same as Fig. 5c, d. **d,** Quantification of sIPSC amplitude and frequency between groups. Each bar graph represents mean ± SEM. Each dot represents one cell. *$p < 0.05$, ***$p < 0.005$. Significance was determined by Kruskal-Wallis test with post-hoc Dunn's test. See Supplementary Data 13 for details of the statistical analysis. Source data are provided as a Source Data file.

Supplementary Fig. 15

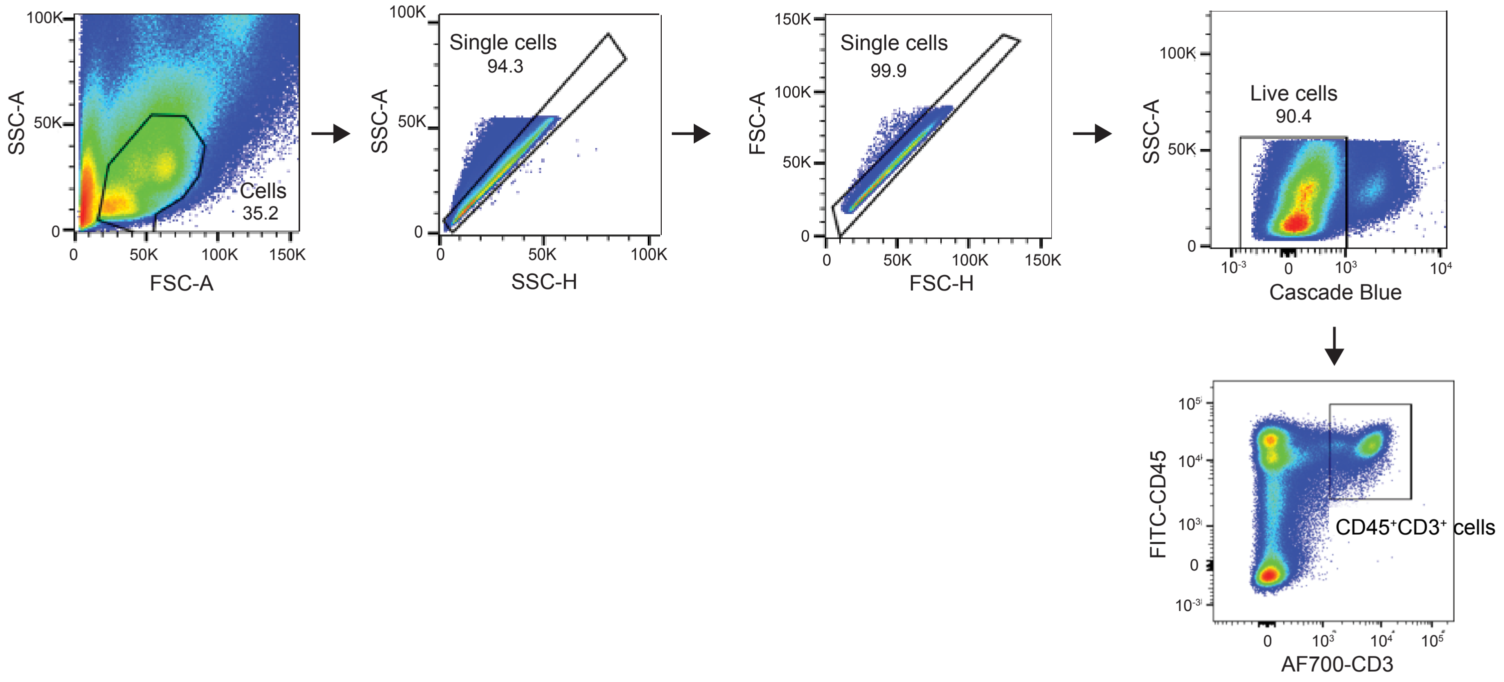

Supplementary Fig. 15: Gating strategies for flow cytometry data.

Density plots showing gating strategies used to analyze the data for **Fig. 8f**. Percentages shown in the plots are calculated based on the parent gate.

Uncropped gel images for Figures

Fig. 1f

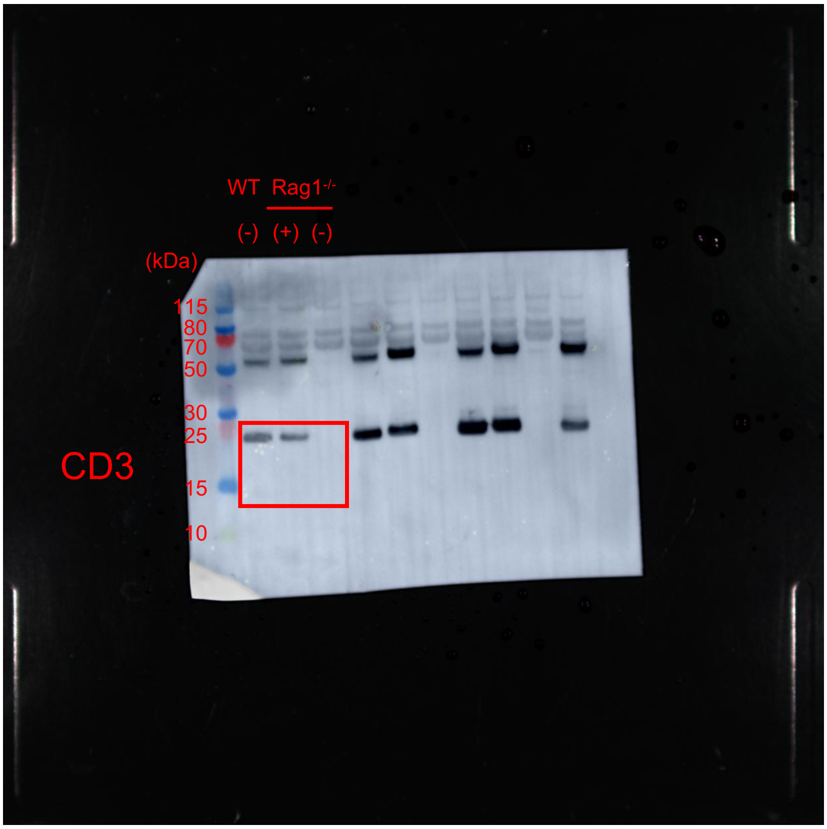

Supplementary Fig. 1c

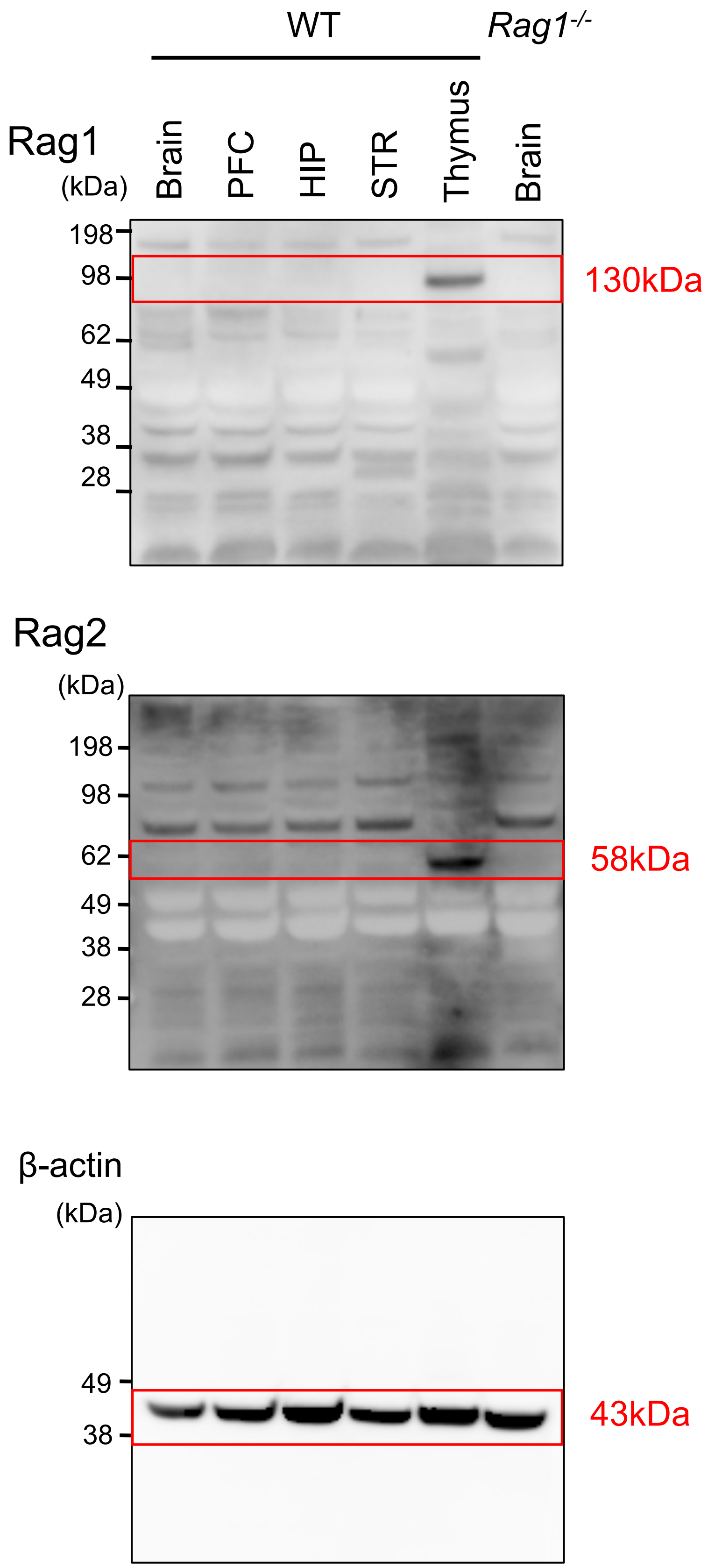

Supplementary Fig. 1d

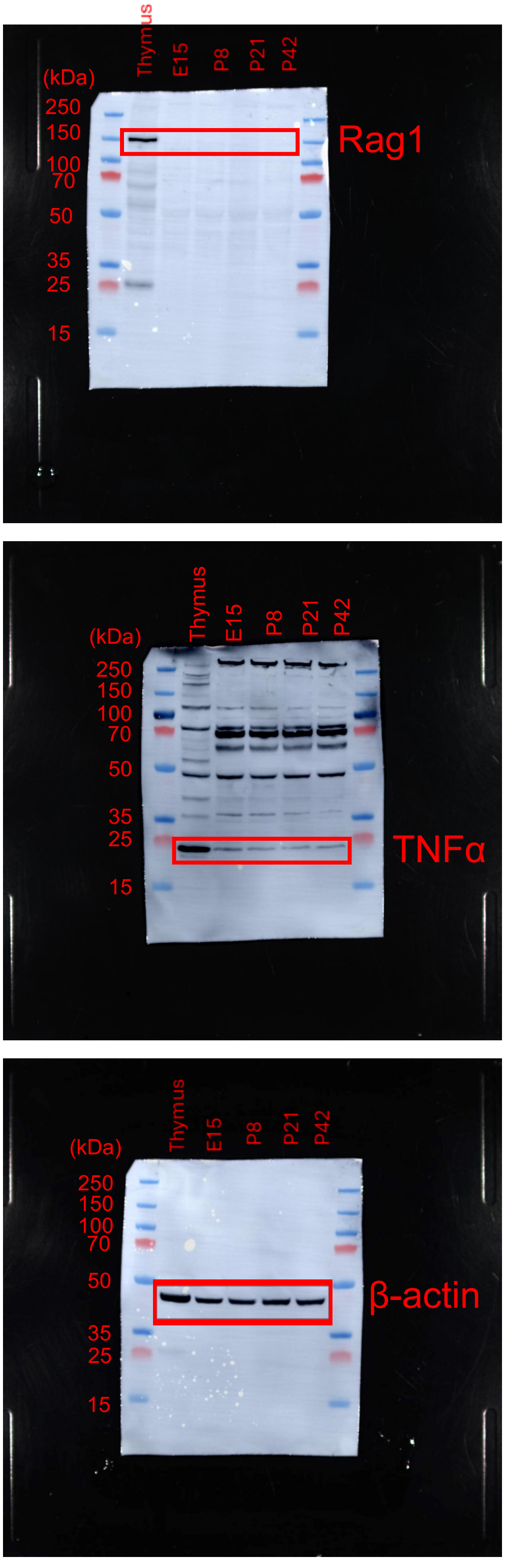

Supplementary Fig. 3b

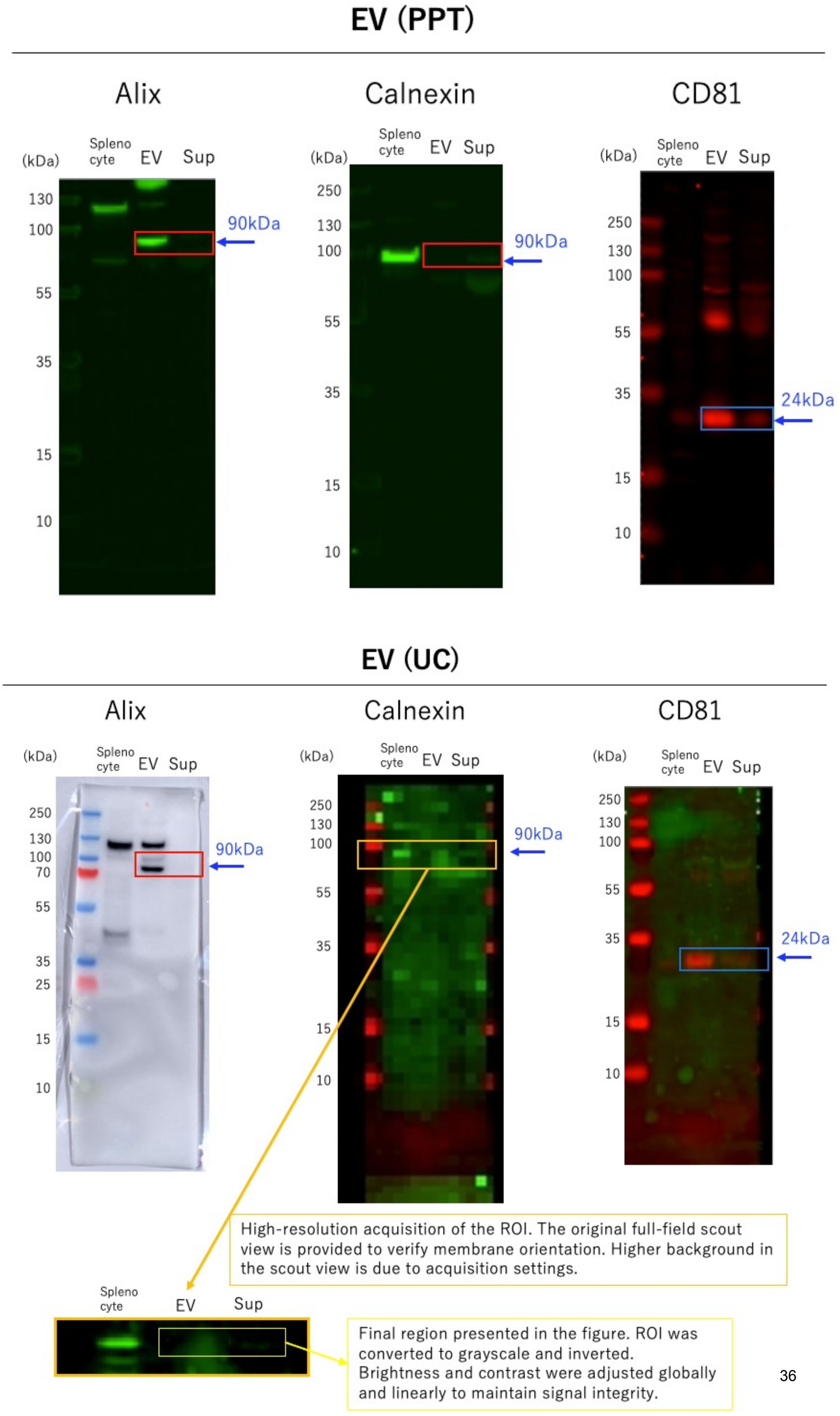

Supplementary Fig. 3f

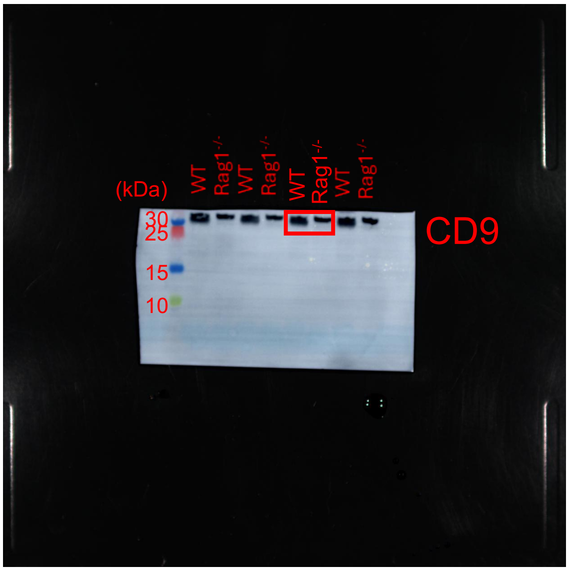

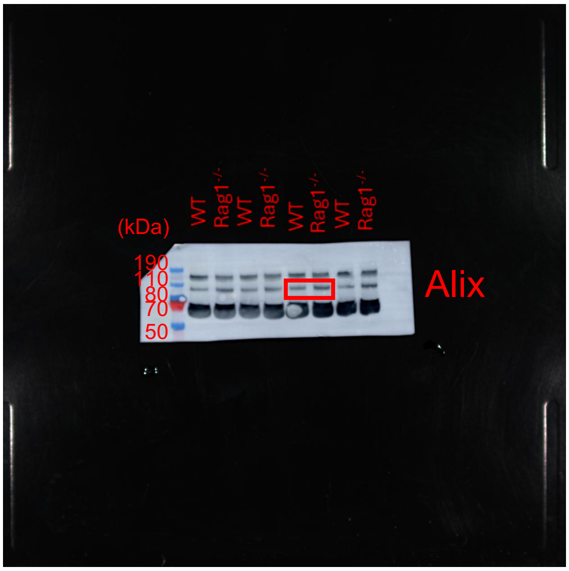

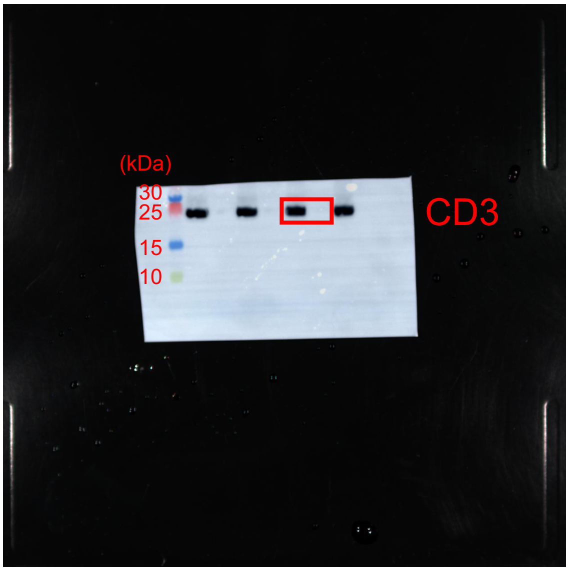

Supplementary Fig. 13a

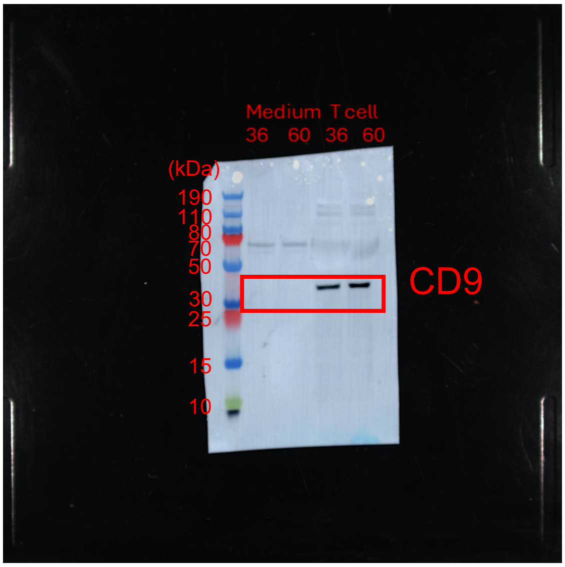

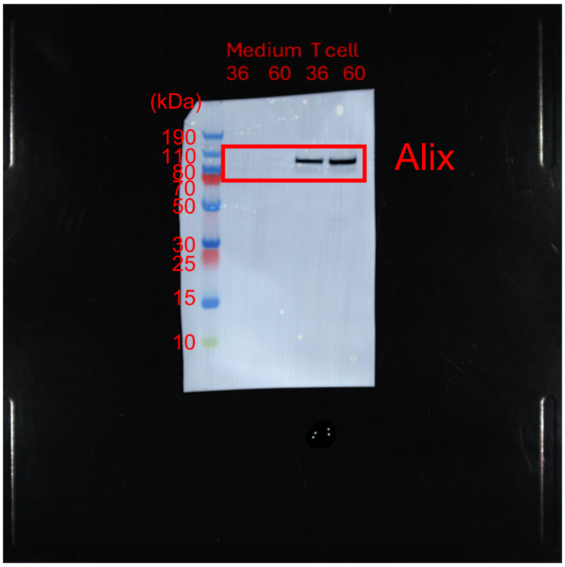

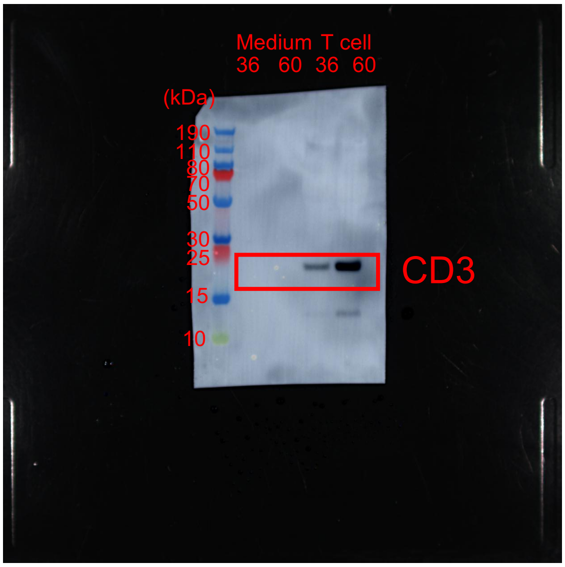

Supplement: Supplementary file 1 — Supplementary Information [file 41467_2026_70469_MOESM1_ESM.pdf]
