# Supplementary material for: Circulating extracellular vesicle microRNAs mediate immune modulation of social behavior in male mice
Source: Nat Commun. 2026 Apr 3;17:4762. doi: 10.1038/s41467-026-70469-1 (PMC13216276; doi:10.1038/s41467-026-70469-1)
Supplement: Supplementary file 2 — Description of Additional Supplementary Files [file 41467_2026_70469_MOESM2_ESM.docx]

**Description of Additional Supplementary Data**

**Supplementary Data 1.** List of differentially expressed miRNAs (adjusted p <0.05) between EVs from *Rag1*^-/-^ mice injected with medium (miRKOmed) or WT T cells (miRKOTcell).

**Supplementary Data 2.** List of differentially expressed miRNAs in the mice with sociability deficit.

**Supplementary Data 3.** List of target genes for the miRNAs differentially expressed between *Rag1*^-/-^ mice injected with medium (miRKOmed) and *Rag1*^-/-^ mice injected with WT T cells (miRKOTcell) from mirTarBase.

**Supplementary Data 4.** GO analysis data for the target genes of the miRNAs differentially expressed between *Rag1*^-/-^ mice injected with medium (miRKOmed) and *Rag1*^-/-^ mice injected with WT T cells (miRKOTcell) (Metascape, q-value < 0.05).

**Supplementary Data 5.** List of genes whose expression was upregulated in the frontal cortex of the *Rag1*^-/-^ mice injected with medium compared with WT mice injected with medium (adjusted p-value <0.05).

**Supplementary Data 6.** List of genes whose expression was downregulated in the frontal cortex of the *Rag1*^-/-^ mice injected with medium compared with WT mice injected with medium (adjusted p-value <0.05).

**Supplementary Data 7.** List of genes whose expression was upregulated in the frontal cortex of the *Rag1*^-/-^ mice injected with medium compared to the *Rag1*^-/-^ mice injected with WT bEV (adjusted p-value <0.05).

**Supplementary Data 8.** List of genes whose expression was downregulated in the frontal cortex of the *Rag1*^-/-^ mice injected with medium compared to the *Rag1*^-/-^ mice injected with WT bEV (adjusted p-value <0.05).

**Supplementary Data 9.** GO analysis data of 1280 genes whose expression were altered in the *Rag1*^-/-^ mPFC and restored by WT bEV injection (Metascape, q-value < 0.05).

**Supplementary Data** **10.** GO analysis data of 755 overlaps (PANTHER Overrepresentation Test)

**Supplementary Data** **11.** Extended list of predicted target genes for the miRNAs differentially expressed between *Rag1*^-/-^ mice injected with medium (miRKOmed) and WT T cells (miRKOTcell) from TargetScan 8.0.

**Supplementary Data 12.** GO pathways related to GABAergic neurons in 755 overlapped genes

**Supplementary Data** **13.** Details of statistical analyses used in this study.
