# Supplementary material for: Circulating extracellular vesicle microRNAs mediate immune modulation of social behavior in male mice
Source: Nat Commun. 2026 Apr 3;17:4762. doi: 10.1038/s41467-026-70469-1 (PMC13216276; doi:10.1038/s41467-026-70469-1)
Supplement: Supplementary file 4 — Reporting Summary [file 41467_2026_70469_MOESM4_ESM.pdf]

nature portfolio

Corresponding author(s): Shin-ichi Kano

Last updated by author(s): Feb 9, 2026

# Reporting Summary

Nature Portfolio wishes to improve the reproducibility of the work that we publish. This form provides structure for consistency and transparency in reporting. For further information on Nature Portfolio policies, see our Editorial Policies and the Editorial Policy Checklist.

## Statistics

For all statistical analyses, confirm that the following items are present in the figure legend, table legend, main text, or Methods section.

| n/a | Confirmed | |
|---|---|---|
| [ ] | [x] | The exact sample size (*n*) for each experimental group/condition, given as a discrete number and unit of measurement |
| [ ] | [x] | A statement on whether measurements were taken from distinct samples or whether the same sample was measured repeatedly |
| [ ] | [x] | The statistical test(s) used AND whether they are one- or two-sided<br>*Only common tests should be described solely by name; describe more complex techniques in the Methods section.* |
| [x] | [ ] | A description of all covariates tested |
| [ ] | [x] | A description of any assumptions or corrections, such as tests of normality and adjustment for multiple comparisons |
| [ ] | [x] | A full description of the statistical parameters including central tendency (e.g. means) or other basic estimates (e.g. regression coefficient) AND variation (e.g. standard deviation) or associated estimates of uncertainty (e.g. confidence intervals) |
| [ ] | [x] | For null hypothesis testing, the test statistic (e.g. *F*, *t*, *r*) with confidence intervals, effect sizes, degrees of freedom and *P* value noted<br>*Give P values as exact values whenever suitable.* |
| [x] | [ ] | For Bayesian analysis, information on the choice of priors and Markov chain Monte Carlo settings |
| [x] | [ ] | For hierarchical and complex designs, identification of the appropriate level for tests and full reporting of outcomes |
| [ ] | [x] | Estimates of effect sizes (e.g. Cohen's *d*, Pearson's *r*), indicating how they were calculated |

*Our web collection on statistics for biologists contains articles on many of the points above.*

## Software and code

Policy information about availability of computer code

| | |
|---|---|
| Data collection | Zen software (Zeiss) -- Confocal microscopy image data<br>Accuri C6 software, CellQuest, FACSDiva (BD Biosciences) -- Flow cytometry data<br>ImageQuant LAS 4000 Control Software, LI-COR Image Studio -- Western blot image data<br>PAS software -- Behavioral video and quantification data (Open field test)<br>Tecnai software -- Transmission Electron Microscopy data<br>NanoSight software, Zetaview software -- Nanoparticle tracking assay<br>SutterPatch software, based on IgorPro -- Electrophysiology data |
| Data analysis | ImageJ/Fiji (ver. 2.3.0/1.53f51) -- immunohistochemical analyses and quantification<br>FlowJo -- flow cytometry data analysis<br>STAR (ver. 2.7.5c), HTSeq-count (ver. 0.12.3), DESeq2 (ver. 1.28.1), Metascape, miRBase (ver. 21) -- RNA-seq data analysis<br>GraphPad Prism (ver. 7, 8, 9; GraphPad Software, Inc), R (ver. 4.1.0), SPSS (ver. 29) and Python (ver. 3.8.5) -- statistical analysis<br>SutterPatch -- electrophysiological data (action potentials and spontaneous currents) |

For manuscripts utilizing custom algorithms or software that are central to the research but not yet described in published literature, software must be made available to editors and reviewers. We strongly encourage code deposition in a community repository (e.g. GitHub). See the Nature Portfolio guidelines for submitting code & software for further information.

## Data

Policy information about availability of data

All manuscripts must include a data availability statement. This statement should provide the following information, where applicable:

- Accession codes, unique identifiers, or web links for publicly available datasets
- A description of any restrictions on data availability
- For clinical datasets or third party data, please ensure that the statement adheres to our policy

The raw bulk RNA-sequencing data will be available at the Gene Expression Omnibus (GEO) before publication.

## Research involving human participants, their data, or biological material

Policy information about studies with human participants or human data. See also policy information about sex, gender (identity/presentation), and sexual orientation and race, ethnicity and racism.

| | |
|---|---|
| Reporting on sex and gender | N/A |
| Reporting on race, ethnicity, or other socially relevant groupings | N/A |
| Population characteristics | N/A |
| Recruitment | N/A |
| Ethics oversight | N/A |

Note that full information on the approval of the study protocol must also be provided in the manuscript.

# Field-specific reporting

Please select the one below that is the best fit for your research. If you are not sure, read the appropriate sections before making your selection.

[x] Life sciences [ ] Behavioural & social sciences [ ] Ecological, evolutionary & environmental sciences

For a reference copy of the document with all sections, see nature.com/documents/nr-reporting-summary-flat.pdf

# Life sciences study design

All studies must disclose on these points even when the disclosure is negative.

| | |
|---|---|
| Sample size | Sample sizes are determined based on previously published experiments or effect size calculation of pilot data. |
| Data exclusions | Pre-established exclusion criteria were applied to behavioral assay in order not to analyze the data from the mice who could not complete tasks (e.g., immobile throughout the session, aggressive interactions between the test and stimulus mice). For eletrophysiological data, patched cells were excluded when displaying variation of baseline values >20% across the experiment duration, and when membrane resistances were too low (< 100 MOhms). |
| Replication | All the experiments were conducted with at least 3 biological replicates as shown and described in each Figure and its legend. |
| Randomization | Mice were randomly assigned into experimental and control groups where applicable. |
| Blinding | No blinding method was used. Blinding was not relevant in this study because all data were obtained through utomated or quantitative analyses that eliminate observer bias. |

# Reporting for specific materials, systems and methods

We require information from authors about some types of materials, experimental systems and methods used in many studies. Here, indicate whether each material, system or method listed is relevant to your study. If you are not sure if a list item applies to your research, read the appropriate section before selecting a response.

## Materials & experimental systems

| n/a | Involved in the study | |
|---|---|---|
| | x | Antibodies |
| x | | Eukaryotic cell lines |
| x | | Palaeontology and archaeology |
| | x | Animals and other organisms |
| x | | Clinical data |
| x | | Dual use research of concern |
| x | | Plants |

## Methods

| n/a | Involved in the study | |
|---|---|---|
| x | | ChIP-seq |
| | x | Flow cytometry |
| x | | MRI-based neuroimaging |

# Antibodies

Antibodies used

Western blot
rabbit anti-CD9 (1:1,000, #ab92726, Abcam, RRID: AB_10561589)
rabbit anti-Alix (1:1,000, #ab186429, Abcam, RRID: AB_2754981)
rabbit anti-Calnexin (1:1,000, #ADI-SPA-860-D, Enzo Life Science, RRID: AB_10616095)
mouse anti-CD3ε (1:1,000, #362701, BioLegend, RRID: AB_2563713)
armenian hamster anti-CD3ε (1:1,000, #100302, BioLegend, RRID: AB_312667)
mouse anti-CD81 (1:1,000, #sc-166029, Santa Cruz Biotechnology, RRID: AB_2275892)
armenian hamster anti-mouse CD81(1:1,000, #sc-18877, Santa Cruz Biotechnology, RRID: AB_627194)
IRDye 680RD Donkey anti-mouse IgG(H + L) (1:10,000, LI-COR Biosciences #926-68072, RRID: AB_10953628)
IRDye 800CW Donkey anti-rabbit IgG(H+L) (1:10,000, LI-COR Biosciences #926-32213, RRID: AB_621848)
rabbit anti-Rag1 (1:1,000, #sc-5599, Santa Cruz Biotechnology, RRID: AB_2300670)
mouse anti-Rag1 (Proteintech #68591-1-Ig, RRID: AB_3085290)
goat anti-Rag2 (1:1,000, #sc-7623, Santa Cruz Biotechnology, RRID: AB_2175836)
mouse anti-β-actin (1:5,000, #sc-47778, Santa Cruz Biotechnology, RRID: AB_626632)
rabbit anti-TNFα (1:1,000, Proteintech #17590-1-AP, RRID: AB_2271853)

Flow cytometry
rat anti-mouse CD3 Alexa Fluor 488 (1:100, #100210 BioLegend RRID: AB_389301)
rat anti-mouse CD19 PE (1:100, #115508, BioLegend, RRID: AB_313643)
rat anti-mouse B220 Alexa Fluor 488 (1:100, #103228, BioLegend, RRID: AB_492874)
rat anti-mouse CD45 FITC (1:100, #110705, BioLegend, RRID: AB_ 313494)
rat anti-mouse CD3 APC (1:100, #100235 BioLegend, RRID: AB_2561455)

Immunohistochemistry
mouse anti-NeuN (1:500, #MAB377, Merck-Millipore, RRID: AB_2298772)
rabbit anti-Iba1 (1:400, #019-19741, Wako Chemicals, RRID: AB_839504)
goat anti-c-Fos (1:500, #sc-52, Santa Cruz Biotechnology, RRID: AB_2106783)
rabbit anti-c-Fos (1:200, #2250 (Clone 9F6), Cell Signaling, RRID: AB_22472111)
mouse anti-CaMKII (1:200, #688602 (Clone 6G9), BioLegend, RRID: AB_2617027)
rabbit anti-RFP (1:1,000, #600-401-379, Rockland, RRID: AB_2209751)
rabbit anti-CaMKIIα (1:100, #20666-1-AP, Proteintech, RRID: AB_2878722)
rabbit anti-PKCε (1:100, #20877-1-AP, Proteintech, RRID: AB_10697812)
mouse anti-CC1 (1:100, #OP80, Calbiochem, RRID: AB_2057371)
rabbit anti-S100β (1:200, #ab868, Abcam, RRID: AB_306716)
mouse anti-NSE (1:500, #66150-1-Ig, Proteintech, RRID: AB_2881546)
mouse anti-Gephyrin (1:100, #147021, Synaptic Systems, RRID: AB_2232546)
rabbit anti-GABAARγ2 (1:100, #AGA-005, Alomone Labs, RRID: AB_2039870)

Validation

All antibodies are from commercially available sources and have been validated from the manufacturer with supporting information on the manufacture's websites. We used them with the concentrations and conditions described there. For antibodies used in immunohistochemistry and flow cytometry, validation experiments were performed in our laboratory by verifying that staining patterns were in agreement with expected patterns. For antibodies used in Western blot, the expected band sizes were verified.

# Animals and other research organisms

Policy information about studies involving animals; ARRIVE guidelines recommended for reporting animal research, and Sex and Gender in Research

Laboratory animals

Species: Mouse
Strain: C57BL/6 wild-type, Rag1-/- and mTmG mice
Age: 8–12 weeks old
Sex: male

| | |
|---|---|
| Wild animals | N/A |
| Reporting on sex | We used only adult male mice (8-12 weeks old, C57BL/6 background) as described in the manuscript. |
| Field-collected samples | N/A |
| Ethics oversight | All the described procedures were approved the Institutional Animal Care and Use Commmitee at the Johns Hopkins University and the University of Alabama at Birmingham. |

Note that full information on the approval of the study protocol must also be provided in the manuscript.

# Plants

| | |
|---|---|
| Seed stocks | N/A |
| Novel plant genotypes | N/A |
| Authentication | N/A |

# Flow Cytometry

## Plots

Confirm that:

- [x] The axis labels state the marker and fluorochrome used (e.g. CD4-FITC).
- [x] The axis scales are clearly visible. Include numbers along axes only for bottom left plot of group (a 'group' is an analysis of identical markers).
- [ ] All plots are contour plots with outliers or pseudocolor plots.
- [x] A numerical value for number of cells or percentage (with statistics) is provided.

## Methodology

| | |
|---|---|
| Sample preparation | Sample preparation procedures are described in the method section. |
| Instrument | BD FACS Calibur, Accuri C6, BD LSRII |
| Software | Accuri C6 software, CellQuest, FACSDiva (BD Biosciences) -- Data acquisition<br>FlowJo -- Data analysis |
| Cell population abundance | No sorting was conducted. |
| Gating strategy | Lymphocyte populations were gated on FSC/SSC plots and doublets were removed on FSC-A/FSC-H plot. Additional information can be provide if necessary. |

- [ ] Tick this box to confirm that a figure exemplifying the gating strategy is provided in the Supplementary Information.
